# Update on the Notochord Including its Embryology, Molecular Development, and Pathology: A Primer for the Clinician

**DOI:** 10.7759/cureus.1137

**Published:** 2017-04-04

**Authors:** Tushar Ramesh, Sai V Nagula, Gabrielle G Tardieu, Erfanul Saker, Mohammadali Shoja, Marios Loukas, Rod J Oskouian, R. Shane Tubbs

**Affiliations:** 1 Neurology, University of Alabama at Birmingham; 2 Department of Anatomical Sciences, St. George's University School of Medicine, Grenada, West Indies; 3 Neurological Surgery, University of Alabama at Birmingham; 4 Swedish Neuroscience Institute; 5 Neurosurgery, Seattle Science Foundation

**Keywords:** notochord, nucleus pulposus, chordoma, spine, embryology, development

## Abstract

The notochord is a rod-like embryological structure, which plays a vital role in the development of the vertebrate. Though embryological, remnants of this structure have been observed in the nucleus pulposus of the intervertebral discs of normal adults. Pathologically, these remnants can give rise to slow-growing and recurrent notochord-derived tumors called chordomas. Using standard search engines, the literature was reviewed regarding the anatomy, embryology, molecular development, and pathology of the human notochord. Clinicians who interpret imaging or treat patients with pathologies linked to the notochord should have a good working knowledge of its development and pathology.

## Introduction and background

**Figure 1:**
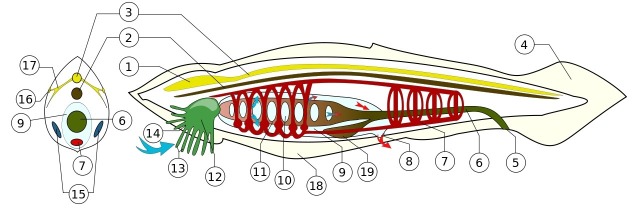
Anatomy of Amphioxus Illustrating the Typical Components of all Chordates 1 = bulge in spinal cord ("brain") 2 = notochord 3 = dorsal nerve cord 4 = postanal tail 5 = anus 6 = digestive canal 7 = circulatory system 8 = atriopore 9 = space above pharynx 10 = pharyngeal slit 11 = pharynx 12 = vestibule 13 = oral cirri 14 = mouth 15 = gonads 16 = photo sensor 17 = nerves 18 = metapleural fold 19 = hepatic caecum

The notochord, namesake of the phylum chordata (Figure 1), plays a central role in vertebrate development. It is most prominent in the first trimester, wherein it guides the folding of the embryo and regulates the differentiation and maturation of surrounding tissues. It is a transient embryologic entity in humans, thought to be completely absent or present in minute quantities, within the nucleus pulposus of intervertebral discs in adults. This entity becomes clinically significant when notochordal remnants give rise to slow-growing and oft-recurring neoplasms known as chordomas. We will discuss the historical discovery of the notochord and its role in embryogenesis, as well as associated molecular signaling pathways, pathology, and treatments for such pathology in this review.

### History

The first sign of a notochord in humans was discovered by Virchow and Luschka in 1857 when they found “vacuolated cells” during autopsies [1]. Through later discoveries, it was found that these cells had a notochordal origin [2]. 

Notochords have long been regarded as the precursors to the spine. They provided the backbones to the earliest chordate ancestors and are now one of the defining characteristics of the phylum chordata. Today our knowledge of the notochord and its impact on embryology and pathology is just beginning to emerge. 

## Review

### Embryology

**Figure 2:**
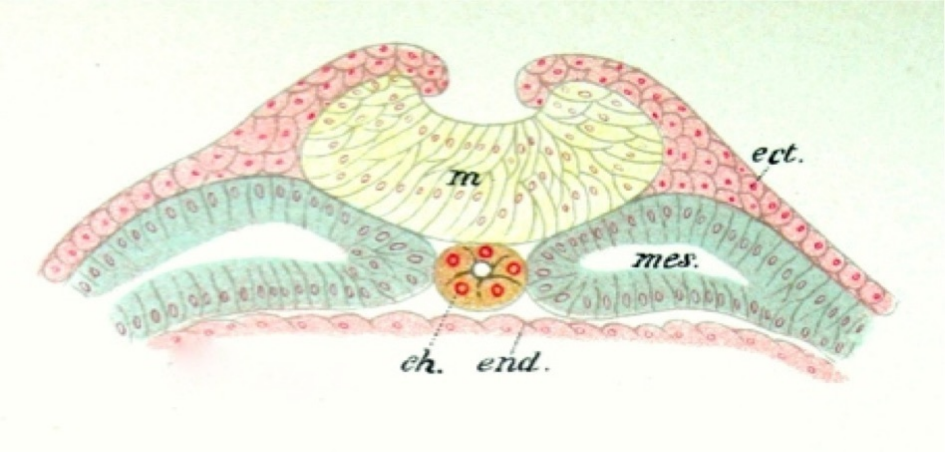
Schematic of the Amphibian Notochord (ch) and Related Germ Cell Layers (From Jakob’s Atlas of the Nervous System, 1901) m - neural tube ect. - ectoderm mes. - mesoderm end. - endoderm

Just before week three of the human embryo development, gastrulation occurs. Around this time, the primitive streak forms. The primitive streak is a cellular structure that starts at the caudal end of the embryo and ends with the primitive node, which is towards the cranial end. Some mesenchymal cells derived from this primitive node migrate cranially, usually around day 19 of development, giving rise to the notochordal process. By day 23, this notochordal process merges with the endodermal cells, forming the notochordal plate, which will subsequently form into the notochord by day 25 [3-4] (Figure 2).

The notochord is widely regarded to have two main roles in embryology: 1. Secreting signaling molecules (namely hedgehog proteins, such as sonic hedgehog (Shh)) to promote the development of the surrounding tissue and 2. Providing structural support to the developing embryo, before it eventually contributes to the formation of the nucleus pulposus regions of the intervertebral discs [3,5].

### Notochord in embryological development

**Figure 3:**
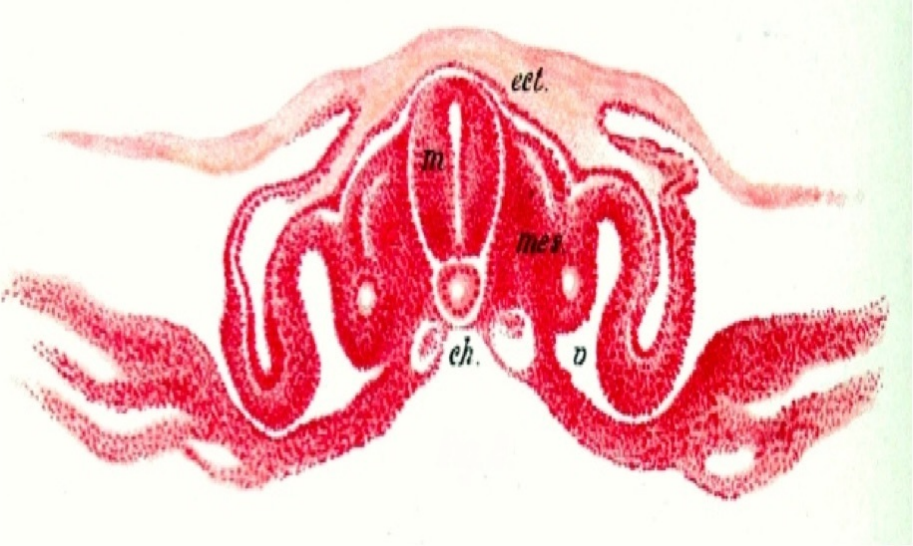
Further Development of the Chick Notochord (ch) and Illustration of the Neural Tube (m), and Overlying Surface Ectoderm (ect) (Jakob’s Atlas of the Nervous System, 1901) mes. - mesoderm

One of the notochord’s most important roles in embryonic development is its patterning of the neural tube (Figure 3). The neural tube arises from neuroepithelial cells and is the precursor to the vertebrate nervous system, including the spinal cord and the brain. The notochord secretes the signaling protein, Shh, which instructs the surrounding cells to specialize. It is at this point that a distinction is made between the dorsal and ventral sides of the neural tube [6]. Sensory neurons arise from the dorsal side, whereas motor neurons arise from the ventral side. Through experiments, it was determined that Shh is the signal that induces this differentiation [7]. This entire process happens between day 23 and day 25 of embryonic development. Along the ventral midline of the neural tube, the floor plate grows from more neuroepithelial cells, due to continued Shh signals from the notochord. The floor plate is an essential structure of the neural tube that promotes further neural cell differentiation and axon guidance in the vertebrate nervous system [8]. While signals from the notochord are essential for the primary formation of the floor plate, the floor plate subsequently releases its own signals to promote the further patterning of the neural tube with Shh as well as other hedgehog class proteins.

Another organ the notochord is thought to have a role in is the development of the pancreas. A series of experiments found that the repression of Shh in dorsal endodermal cells, rather than the expression of it, is what allows for the development of endoderm into the pancreatic tissue [9]. The notochord is thought to secrete signals (activin and fibroblast growth factor 2 (FGF2)) that hinder the production of Shh in the endoderm. Instead, these notochordal signals cause the expression of pancreatic developmental genes in the endodermal cells, namely the transcription factors pancreatic duodenal homeobox 1 (PDX1) and islet1 (ISL1) [9].

### Intervertebral disc (IVD) formation and bone morphogenesis

The coordinated breakdown of the notochord begins in week five of embryologic development. First, the perinotochordal basement membrane forms in the alternating regions of condensed and loose cells. Condensed regions develop into vertebral bodies, while the looser regions become the anulus fibrosus. A majority of notochordal tissue condenses to form the nucleus pulposus [10], comprising the interior of the anulus fibrosus. It was suggested by Walmsley [10] that cells located in the notochord form the nuclei pulposi, and 55 years later, in the mid-2000s, fate-mapping experiments performed using the mouse model system showed that all nuclei pulposi cells came from the cells in the notochord [11-13]. Cells from the surrounding anulus fibrosis or end plates were not seen to migrate to the nuclei pulposi, thus in agreement with the notion that the notochord is the only source of cells found in the nuclei pulposi throughout the life of the mouse [11-13]. From this experiment, it is proposed that a similar mechanism occurs in humans. Studies have shown that the formation of the vertebrae and the disc occur simultaneously [10,12,14]. One model, the "pressure" model, proposed that while mesenchymal cells are condensed into the vertebrae, notochord cells are “squeezed” and “pushed” into areas of the vertebral column where the discs are formed [10,12]. In this model, it is suggested that the condensing vertebrae play a role by pushing the notochord cells into areas of disc formation [12]. Another model, the “repulsion/attraction” model suggested that areas of disc formation may express attractant molecules, which are detected by notochord cells resulting in these cells gathering between the vertebrae. Alternatively, the areas of vertebrae formation of the vertebral column can emit repulsive signals excluding notochord cells and cause them to occupy the space between the forming vertebrae [12]. There are many signaling pathways that may play a role such as eph/ephrin or robo/slit [12]. This notochordal tissue will go on to develop into the mature intervertebral disc. However, small amounts of notochord tissue are thought to persist in the center of vertebral bodies and the nucleus pulposus, into early childhood and adulthood.

Vertebral body formation is not completely understood in humans. In teleosts, studies have shown that concentric rings of bone are induced in segmented regions within the notochordal sheath that give rise to vertebral bodies. These bony regions, or centra, form due to an inherent periodicity of the notochord, and can be completely absent if specific regions of the notochord are ablated [5]. Other studies demonstrate the importance of notochord vacuoles in the structural integrity of vertebral bodies [5]. One study of zebrafish correlated the location of fragmented vacuoles with the location of structural defects, during spinal ossification [15]. This suggests that congenital malformations of the vertebral column may be a result of trauma or injury to notochordal cells in early embryological development [5].

### Postnatal life

The fate of notochordal cells within the nucleus pulposus has only recently been elucidated. In humans, within the first decade of life, nucleus pulposus cells go through radical changes unlike the cells of the anulus fibrosus and cartilage-end plates. Immediately after birth in most vertebrates, including mice and humans, large vacuolated cells are progressively lost and the nucleus pulposus becomes occupied by small cartilage-like nucleus pulposus cells [16-18]. These large vacuolated notochord cells are believed to play a role in the maintenance of the nucleus pulposus as the loss of these cells have been associated with the beginning of disc degeneration [16-17,19-20]. Studies of disc degeneration in rodents and canines led some to contend that notochordal cell populations decline after birth and are replaced by chondrocyte-like cells of a non-Shh producing lineage. There has been some debate regarding the origin of the cartilage-like cells of the nucleus pulposus. Recent cell ontology studies involving the recombinase gene, cre, have shown that definitive markers of Shhcre+ notochord cells persist throughout the nucleus pulposus, providing new evidence that those chondrocyte-like cells are indeed derived from Shh-expressing notochord cells [13,17]. Prior to this breakthrough study, it wasn’t possible to rule out the possibility that chondrocyte-like cells were descendants of the surrounding Shh-negative mesenchyme, including cells of the anulus fibrosus and cartilaginous endplates.

It was thought that these cells originated from the mesenchymal surrounding cartilage-end plates [21] or the perichondrium at the border of the IVD [22] that migrated to the nucleus pulposus. Notochord cells were thought to control mesenchymal cell migration, activate matrix synthesis, and then go through apoptosis or necrosis [17,23-24]. Contrary to this, more current studies have proposed that the notochord cells act as progenitors of the nucleus pulposus and go through final differentiation to produce cartilage-like nucleus pulposus cells (notochord cell maturation model) [17,20,25-26]. McCann and Séguin [17], as well as others, carried out lineage-tracing studies in mice that showed that all cells of the nucleus pulposus are notochord-derived during normal development and aging. In a study to examine the cellular composition of the nucleus pulposus during intervertebral disc degeneration, bone marrow stromal cells (BMSC) were marked with green fluorescent protein (GFP) and no BMSC were found in the nucleus pulposus, thus supporting the notochord cell maturation model. “Nucleus pulpocytes” has been the name suggested by recent publications for these mature cells within the nucleus pulposus to emphasize their distinguishable developmental origin and function [9,17].

Notochord cells within the postnatal intervertebral discs are believed to preserve their role as vital signaling effectors regulating intervertebral disc cell function. Notochord secreted factors have also been shown to protect nucleus pulposus cells from the degradative effects of cytokine exposure and inhibit nucleus pulposus cell death and apoptosis [17,27].

The nucleus pulposus of the mature intervertebral disc differs vastly from that of the neonate. In the neonate, the nucleus pulposus has relatively few extracellular aggrecans and other proteoglycans. Over time, the disc develops a higher and higher proteoglycan matrix-to-cell ratio [10,14,17]. In an adult, the nucleus pulposus is found within a hypoxic, hypertonic environment. It is avascular and depends on diffusion for its nutrients, relying solely on the glycolytic pathway for metabolism [14,17]. It is thought that various transcription factors upregulated in the acidification of the nucleus pulposus, including tonicity-responsive binding-protein (tonEBP), are implicated in the survival and differentiation of notochordal cells [28].

It is thought that alterations to the vascular supply of the anulus fibrosus play a role in the onset of degenerative disc disease. Even a transient decrease in oxygen tension would have significant effects in the already hypoxic microenvironment of the anulus fibrosus and could lead to a failure in notochordal progenitor cell activation. This process could compromise the intervertebral disc’s ability to withstand biomechanical forces and limit the spine’s range of motion and set into motion other biochemical processes associated with the onset of degenerative disc disease [18].

**Figure 4:**
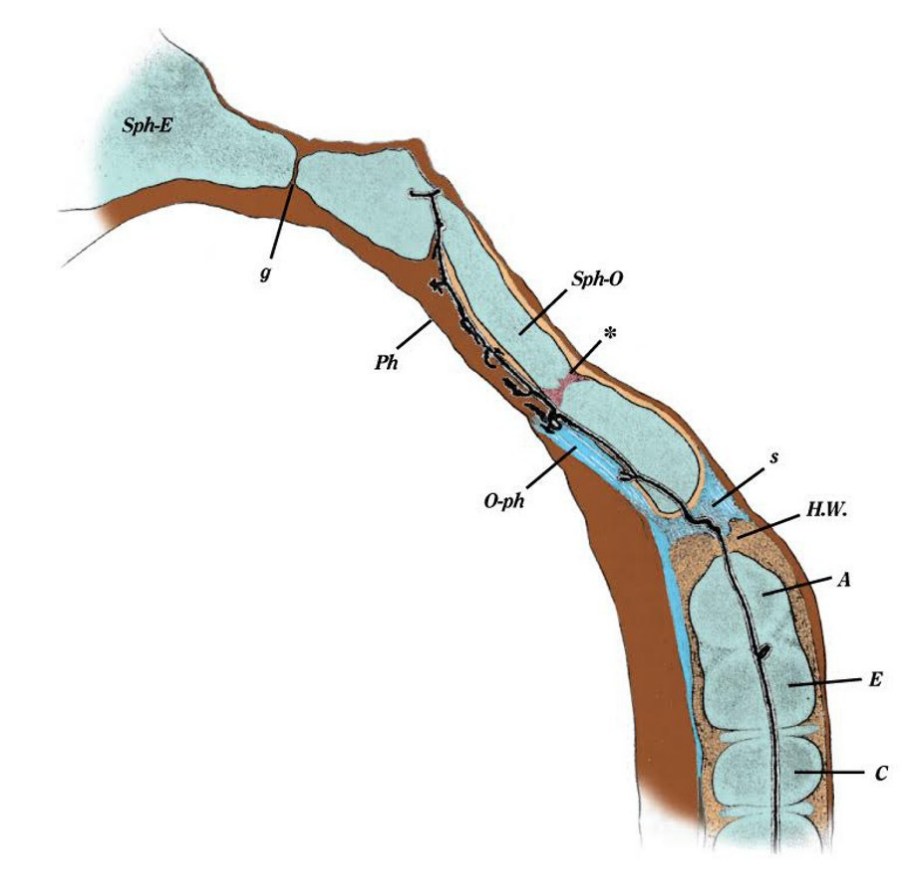
Sagittal Section Through C2 Vertebra and Skull Base Note the position of the notochord (small black tube) as it ascends (Froriep).

**Figure 5:**
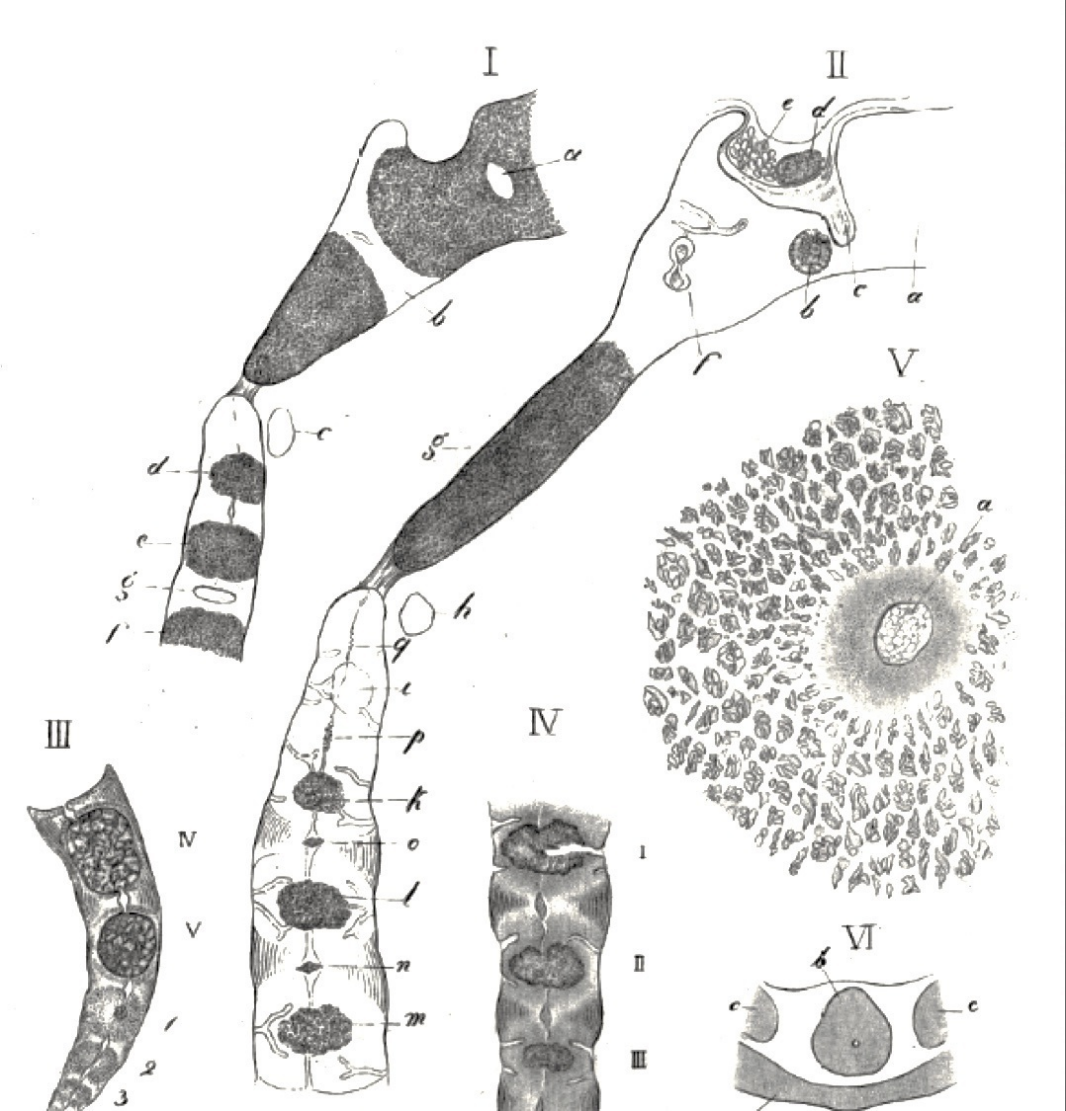
Composition Image From Müller’s 1858 Ueber das Vorkommen von Resten der Chorda dorsalis bei Menschen nach der Geburt und uber ihr Verhaltniss zu den Gallertgeschwulsten am Clivus I. Median cut through the skull base and the three upper cervical vertebrae of a one-year-old child. a. Remains of cartilage in sphenoid. b. Synchondrosis between sphenoid and occipital bone. A ‘rest’ of the notochord is visible at this site. c. Anterior arch of the atlas. d-f. Osseous cores of the odontoid process and bodies of C2 and C3. g. Cave in the synchondrosis between the second and third cervical vertebrae with a notochord cell rest. II. Median cut from a 6 ½-inches human embryo. a. Cartilage of the sphenoid. b. Bony core. c. Cone of the perichondrium that penetrates near the cartilage. d. Front. e. Posterior lobe of the pituitary gland. f. Cave of the cartilage that contains a ‘rest’ of the notochord. In front, is a triangular narrow duct that probably communicates with this cave. g. Ossified part of the basilar part of the occipital bone. h. Anterior arch of the atlas. i. Bone of odontoid process of C2. k-m. Bony core for the bodies of the second, third, and fourth cervical vertebrae. n-o. Notochord remnants. p. Spindle-shaped notochord-remains between body and dens of C2. q. Narrow cave with notochord-remains in the odontoid process. III. Median cut through the lower end of the spine of a newborn child. Note the small notochord part ascending up the middle of the bone. IV. Median cut through the sacrum of a 6 ½-inches human embryo. The four upper sacral vertebrae also noting notochord in the midline. V. Transverse cut through the cartilage of the axis of a newborn. a. Rest of the notochord - here surrounded by a very thick and dark matrix of the cartilage. VI. Transverse cut through the atlas and C2 of a 4 ½-inches human embryo. a. Atlas. b. Dens with notochord remnant.

The cephalad extension of the notochord travels from the odontoid process of the axis into the basiocciput (Figures 4-5), hence the propensity for notochord remnants in this region to give rise to chordomas. These are notochord-derived neoplasms of the bone that are rare and invasive, and account for 20% of primary spine tumors [17]. They are frequently located at the clivus (32%) and the sacrococcygeal region (29%), and less often in the cervical, thoracic, and lumbar vertebrae [7,17]. On the contrary, a recent cadaveric study on the apical ligament of the craniocervical junction, conducted by Fisahn, et al. [29], showed the absence of notochord tissue in the apical ligament. Although the path of this structure is similar to that of the notochord during early development, these results propose that chordoma development from the apical ligament is unlikely, and question the origin of tumors of the craniocervical junction, such as the clivus [29]. Fisahn, et al. [29] state that, in adults, the apical ligament should not be regarded as a notochord remnant. Chordomas are similar to notochord cells in terms of morphology and are produced by physaliferous cells that co-express notochord cell genes as well as brachyury [12,21], and cytokeratins 8, 18, and 19 [17]. In humans, 20% of adult vertebrae were observed to have notochord cell remnants; however, they did not develop into tumors [12,17,30]. It is hypothesized that activation and proliferation of notochord remnants result in chordoma formation.

### Notochord in embryological structure and fate after development

The notochord also plays a crucial role in the structure of a developing embryo. As it is the precursor to the spine, it can be thought of as a transient spine of the embryo, while the actual spinal cord develops from the neural tube [31]. The structure of the notochord resembles that of a stiff, yet flexible rod. On the inside, highly vacuolated cells that are filled with fluid exist, surrounded by a layer of epithelial-like cells. Surrounding this is an extracellular sheath that contains a layer of collagen, which runs parallel to the notochord, and another loosely organized perpendicular matrix. This architecture allows for a rigid outer layer, which counterbalances the hydrostatic pressure coming from the filled inner vacuolated cells, creating a flexible, yet firm rod-like structure [5].

## Conclusions

Even though the notochord is a transient structure, notochordal cells are still present in our bodies. During the late stages of embryonic development, the outer layer of the notochord condenses in areas to form the vertebrae of the spine. Notochordal tissue has been found to exist in the centers of adult intervertebral discs, which are the layers of cartilage in between vertebrae units providing most of the spine’s flexibility. At the center of these intervertebral discs exists the nucleus pulposus, which is what notochord cells condense to form. Many spinal conditions, including intervertebral disc degeneration and chordomas, can be understood with the notion that they may originate from early problems in embryonic notochord structure and function.
